# Palm Oil in Lipid-Based Formulations and Drug Delivery Systems

**DOI:** 10.3390/biom9020064

**Published:** 2019-02-13

**Authors:** Danial Efendy Goon, Siti Hamimah Sheikh Abdul Kadir, Normala Ab Latip, Sharaniza Ab. Rahim, Musalmah Mazlan

**Affiliations:** 1 Department of Biochemistry, Faculty of Medicine, Universiti Teknologi MARA (UiTM), Cawangan Selangor, 47000 Sungai Buloh, Selangor, Malaysia; danialefendy@ymail.com (D.E.G.); drsharaniza@salam.uitm.edu.my (S.A.R.); musalmah6393@salam.uitm.edu.my (M.M.); 2 Institute of Medical Molecular Biotechnology (IMMB), Faculty of Medicine, Universiti Teknologi MARA (UiTM), Cawangan Selangor, 47000 Sungai Buloh, Selangor, Malaysia; 3 Atta-ur-Rahman Institute for Natural Products Discovery (AuRIns), Faculty of Pharmacy, Universiti Teknologi MARA, 42300 Puncak Alam, Cawangan Selangor, Selangor, Malaysia

**Keywords:** palm oil, palm kernel oil, palm fatty acids, medium-chain triglycerides, lipid-based formulation, drug delivery system

## Abstract

Palm oil is natural oil packed with important compounds and fatty acids ready to be exploited in lipid-based formulations and drug delivery. Palm oil and palm kernel oil contain long-chain and medium-chain triglycerides, respectively, including phytonutrients such as tocotrienol, tocopherol and carotenes. The exploitation of these compounds in a lipid-based formulation would be able to address hydrophobicity, lipophilicity, poor bioavailability and low water-solubility of many current drugs. The utilisation of palm oil as part of the drug delivery system seemed to improve the bioavailability and solubility of the drug, stabilising emulsification of formulation between emulsifier and surfactant, promoting enhanced drug permeability and performance, as well as extending the shelf-life of the drug. Despite the complexity in designing lipid-based formulations, palm oil has proven to offer dynamic behaviour in providing versatility in drug design, form and delivery. However, the knowledge and application of palm oil and its fractions in lipid-based formulation are scarce and interspersed. Therefore, this study aims to focus on the research and outcomes of using palm oil in lipid-based formulations and drug delivery systems, due to the importance of establishing its capabilities and benefits.

## 1. Introduction

**Figure 1:**
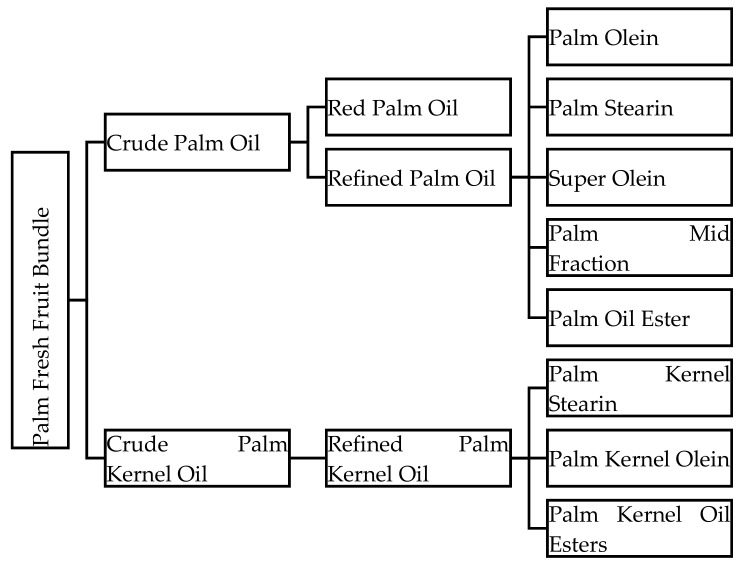
Overview of downstream products of palm oil and its fractions.

The global demand for oil from palm tree *Elaeis guineensis* has increased compared to other types of oil and fats. The increasing demands of palm oil (PO) and palm kernel oil (PKO) are due to increased public reception and appreciation of their potentials and benefits compared to other types of vegetable oils. Both crude palm oil (CPO) and crude palm kernel oil (CPKO) acquired from the palm tree require further refining and processing to produce fractions with refined compositions. Crude palm oil is processed and refined to produce PO while CPKO is refined to produce PKO. Both oils have been utilised in various sectors, primarily in the food industry [1], non-food industrial [2], manufacturing [3] and pharmaceuticals [4]. The part of oil palm fruit known as the mesocarp produces CPO while the oil palm fruit kernel produces CPKO [5]. Downstream products and fractions of palm oil from fresh fruit bundle processing are illustrated in Figure 1. 

The benefits of palm oil as the component in pharmaceutical products and supplements were reported back in 1985 [4]. The study identified and described specific fractions of palm oil and phytonutrients to determine their role in certain blends or mixtures in producing drugs or in pharmaceutical applications. Among the important phytonutrients in palm oil beneficial to pharmaceutical products are vitamin E [6,7], carotenes [8,9] and triglycerides [10]. The concentration of vitamin E (tocotrienol and tocopherol) and carotenes of CPO were found to be higher compared to other natural oils such as corn and olive oil. Tocotrienol and tocopherol are known for their antioxidant activities in reducing radical damages from organs down to the molecular level. The effects of its antioxidant properties were observed in both animal and human studies [11,12]. For many years now, researchers demonstrated that the primary fatty acids (FAs) in palm oil, such as lauric acid, myristic acid and palmitic acid, increase the risk of cardiovascular diseases (CVD) in individuals. However, recent scientific evidence indicates the contrary, whereby palmitic acid in palm oil was shown to increase the level of good cholesterol (high density lipoprotein (HDL)), compared to other saturated fatty acids [13,14,15]. In fact, palm oil has been proven to increase the level of good cholesterol in comparison to the average western dietary fat, which consists of daily consumption of vegetable oils primarily from corn and peanut [16]. Moreover, the effect of palm oil is not limited to increasing total HDL/cholesterol ratio, but able to reduce the plasma cholesterol in individuals who are on a regular Western diet as well [17]. In addition, the antioxidant constituents present in palm oil may even neutralise the content of palmitic acid in palm oil. In another study, the monosaturated fat in palm oil is suggested to lower serum lipid and lipoprotein profiles compared to olive oil, soybean oil and corn oil. The studies above highlighted the capacities and capabilities of palm oil fractions in pharmaceutical products [18,19]. Further identifications and formulations should be investigated in marketing palm oil-based pharmaceutical products as the level of phytonutrients, such as tocotrienol, tocopherol, β-carotenes and fatty acids, are shown to surpass other vegetable oils.

The development of a new drug molecule requires more than at least 15 years of research, and may involve billions of dollars of funding. Hence, the pharmaceutical industry ventures into improving the existing drugs, such as in lipid-based formulations (LBFs) [20]. Lipid-based formulation is an approach to improve oral absorption of a drug and to maintain its supersaturated drug concentration [21,22]. In this approach, the hydrophobic drug is dissolved and emulsified in LBF until absorption has occurred. The primary factor in LBF is the lipid excipients [23,24]. The lipid excipients play the most critical role in formulation performance. For the past 20 years, LBFs have evolved with different ratios of ingredients and compounds. An LBF must be studied and optimised before being tested in animal models or in clinical trials. A pseudoternary diagram is generated from the formulation, and testing is done in in vivo and in vitro models [25,26]. Excipients in LBF from olive and peanut oils have been extensively studied compared to palm oil. However, palm oil showed a promising outcome, and has been shown to possess a dynamic profile based on the pseudoternary diagram, making the oil suitable and stable in drug formulation and delivery [27]. It is not heavily affected by other surfactants in forming micelles and it is possible to improve the formulation [28] compared to other commonly selected oils. The fractions from both CPO and CPKO (Figure 1) can be selected in developing LBFs, rather than only a part from its fruit. Palm oil appeared as a resourceful choice as the excipient in LBFs. Hence, in this study, we provide a comprehensive summary and characterisation of LBFs that employ palm oil as their excipient. This review also focuses on the benefit of using palm oil to obtain LBFs for oral and topical delivery, along with a brief discussion on the future of palm oil as an LBF excipient.

## 2. Composition of Crude Palm Oil (CPO) and Crude Palm Kernel Oil

**Figure 2:**
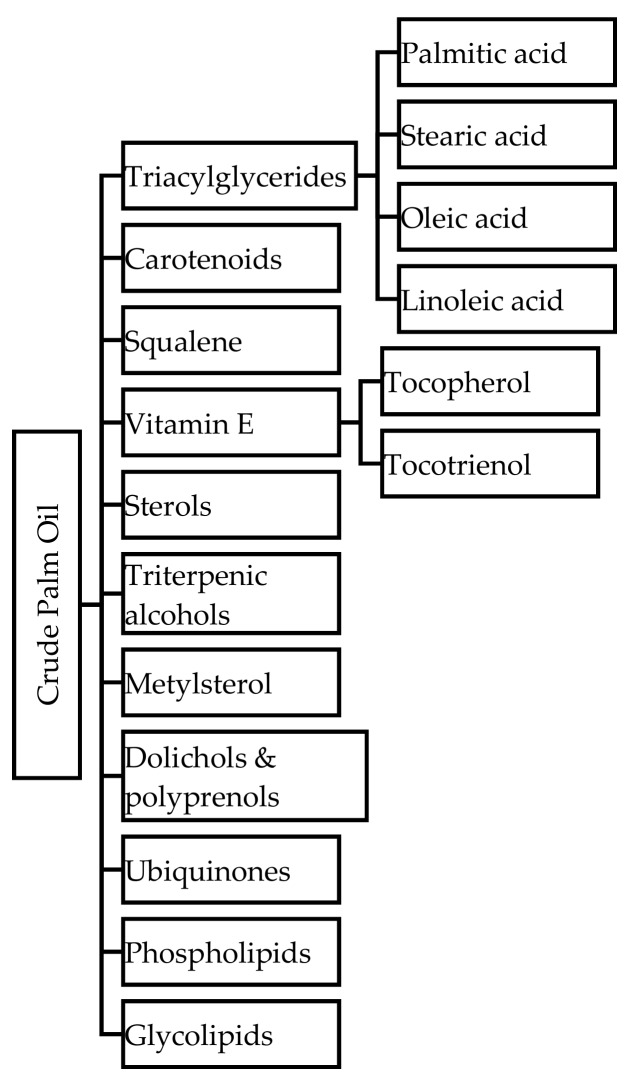
Crude palm oil (CPO) constituents.

Crude palm oil extracted from the mesocarp is mainly composed of triacylglycerides (TAG) [5]. CPO contains 50% saturated and 50% unsaturated FA. The saturated FA distribution in CPO is 94% TAG and 6% diacylglyceride (DAG). The major saturated FAs are represented in the majority by palmitic acid (C_16:0_, 45%) and stearic acid (C_18:0_, 3.5%). Unsaturated FAs consisted of oleic acid (C_18:1_, >59%) and linoleic acid (C_18:2_, >18%) [29]. The minor components in CPO are carotenoids (500–700 ppm), squalene, vitamin E, sterols, triterpenic alcohols, methylsterol, dolichols and polyprenols, ubiquinones, phospholipids and glycolipids [30]. The summary of CPO’s major and minor constituents, described above, is summarised in Figure 2 below. The percentages and types of FAs in palm oil and its fractions is listed in Table 1. 

Despite both CPO and CPKO being derived from the same plant, CPKO possessed different components compared to CPO. First, CPKO contains a much lower percentage of FA. Studies showed CPKO contains about 0.4% to 6.8% of FA [31,32]. However, CPKO is richer in long-chain FA. Its FA is composed of 83% saturated fat high in lauric acid, myristic acid and oleic acid [33]. In terms of unsaturated FA, it is composed of 15% monounsaturated FA and 3% of polyunsaturated FA. The carotene level in CPKO is estimated at only a few ppm. The tocopherol and tocotrienol contents are low as well, estimated at about 30 to 100 ppm. The major isoforms in this oil are α, β, γ-tocopherols and γ-tocotrienol [33,34]. 

The content of refined–bleached–deodorised palm oil (RBD PO) is similar to those in CPO due to limited post-processing modification [5]. Palm olein (PL) and palm stearin (PS) are downstream products from the further fractionation of RBD PO. Due to the unique ratio of saturated and unsaturated TAG, the fractionation process produced both solid and liquid oil, referring to stearin and olein, respectively [35]. A study discriminating the properties of PL and PS was previously carried out [36]. The percentage of TAG in PL retained its properties from RBD PO. The majority of the FAs detected in PL were in the C_18:1_ configuration, signifying oleic acid (43%) followed by 41% of palmitoleic acid (C_16:0_). Linoleic acid (C_18:2_) was also detected at 11% [37]. Tocopherol and tocotrienol are both present in PL at a higher volume compared to RBD PO. The content of FA in PS is high in palmitoleic acid (C_16:0_) between 45% to 57% and oleic acid (C_18:1_), approximately 31% to 40% [22,23,24]. Tocotrienol component in PS is dominated by γ-tocotrienol (49%) followed by α-tocopherol (18%) and α-tocotrienol (17%). However, the content of vitamin E isoforms in PS is one-fourth of those in PL [38].

Double fractionation of olein resulted in the production of super olein. The characteristics of super olein include the absence of trisaturated TAG, a high percentage of monosaturated TAG and low content of disaturated TAG. The FAs present in super olein are mainly long-chain TAG, such as oleic acid (C_18:1_, 47%) and palmitic acid (C_16:0_, 35%). Other FAs include linoleic acid (C_18:2_) and stearic acid. Super olein is 91% TAG and 9% DAG. Palm mid-fraction (PMF), fractions of palm oil produced by a special refining process, possessed a higher percentage of disaturated TAG (75%) and low monosaturated TAG (<30%) [39]. Palmitic acid represents the major FA (58%) in PMF, followed by oleic acid (32%). Other FAs present in PMF in less than 10% are stearic acid, linoleic acid and myristic acid [40]. Lastly, palm oil ester (POE) is acquired by enzymatic alcoholysis of palm oil with the use of oleyl alcohol and catalyst. Palm oil ester exhibited wetting behaviour without the grease properties in RBD PO [41]. Palm oil ester consists of 42% oleyl palmitate (C_34:1_), 32% oleyl oleate (C_36:2_) and 10% oleyl linoleate (C_36:3_). Palm oil ester exhibited a low irritancy score when applied on human skin, making it a favourable ingredient as a topical drug and in cosmetic manufacturing [42].

The percentage of oil content in RBD PKO is approximately 48% [43]. The FA in RBD PKO is mostly lauric acid (C_12:0_) and myristic acid (C_14:0_). A study on the distribution of FA showed that lauric acid comprises about 48% while myristic acid is about 16%, followed by 15% oleic acid. Saturated FA was detected at 82% while monosaturated FA was 15% and polyunsaturated FA was 2.4% [44]. A higher proportion of lauric acid in RBD PKO suggested that this oil is a saturated medium-chain FA oil, while RBD PO is a saturated long-chain FA oil with oleic acid as its major component. Carotenoids were not detected in RBD PKO. However, huge portions of sterols detected were β-sitosterol, stigmasterol and campesterol. The cholesterol level was as low as 14 mg kg^−1^ (1.6%) [33]. 

The FA composition in palm kernel olein (PKL) is dominated by lauric acid (C_12:0_) at 46%. Other FAs detected in PKL were oleic acid (C_18:1_) at 18% and 14% of myristic acid (C_14:0_). Other FAs in a lower proportion, with of less than 10% detected, were caprylic acid, capric acid, palmitic acid, stearic acid, oleic acid and linoleic acid [45]. These values were supported by another study by Dian et al. [46]. In palm kernel stearin (PKS), the major proportion of FA is similar to PKL but at a higher percentage. Lauric acid is approximately 56% and myristic acid is about 21%. Other FAs occurring at less than 10% were identified as caprylic acid, capric acid, palmitic acid and stearic acid [47,48]. Palm kernel oil ester (PKOE) possessed 54% oleyl laurate (C_30:1_), 14% oleyl myristate (C_32:1_), 6% oleyl palmitate (C_34:1_) and 6% oleyl oleate (C_36:2_) [42]. 

As an overview of palm oil constituents, CPO downstream products from fractionation showed an increase in some constituents due to the refining process. For example, the CPO concentration of carotenes and vitamin E are lower compared to RBD PO. The FAs, however, remained in a nearly similar ratio between those two, including other fractions such as PL and PS. CPKO and its fraction contain a lower amount of FA compared to CPO and its fractions. However, as CPKO is refined and has undergone fractionation, the volume of saturated FA increased with the major portion of these FAs in the C_12_ to C_18_ configuration, suggesting they mainly consist of medium-chain triglycerides (MCT), except for PKOE. 

## 3. Triglycerides in Palm Oil and Fractions

Triglyceride (TAG), formed by three FAs and esterified with glycerol, is an active form of storage of fatty acids. The carbon number of fatty acids in TAG can be grouped based on their length as each group possessed its own physiological and metabolic pathways. Fatty acids with 1 to 11 carbon chains are grouped as short-chain triglycerides (SCTs), fatty acids with 7 to 12 carbon chains are known as medium-chain triglycerides (MCTs) and those with more than 13 carbon chains are long-chain triglycerides (LCTs). SCTs include formic acid (C1), acetic acid (C2), propionic acid (c3), butyric acid (C4), isobutyric acid (C4), isovaleric acid (C5) and hexanoic acid (C6). MCTs include enanthic acid (C7), octanoic/caprylic acid (C8), capric acid (C10), undecylic acid (C11) and lauric acid (C12) [50]. Tridecyclic acid (C13), myristic acid (C14), palmitic acid (C16) and stearic acid (C18) are grouped under LCTs. By analysing the major FAs in palm oil and their fractions, they can be classified into either LCTs or MCTs. Crude palm oil contains mainly 59% of oleic acid and 18% linoleic acid, making this oil primarily composed of LCTs. CPKO, however, contains a high level of lauric acid, which is an MCT. RBD PO contains mainly LCTs, whereby the major FAs found are palmitic acid, stearic acid and myristic acid. RBD PL contains a high concentration of LCTs, mainly oleic acid, palmitoleic acid and linoleic acid. Similarly, RBD PS contains the majority of palmitoleic acid and oleic acid. Super olein and PMF contain a high amount of LCTs with a major proportion of oleic and palmitic acid. RBD PKO contains mainly MCTs with 48% lauric acid followed by a small percentage of myristic acid and oleic acid. Similarly, both RBD PKL and PKS contain MCTs, primarily with a high level of lauric acid. 

### 3.1. Medium-Chain Triglycerides and Long-Chain-Triglycerides: Excipients for Lipid-Based Drug Formulation

Medium-chain triglycerides and LCTs have been under heavy investigation for their role of providing benefits and advantages in human metabolism, physiological response and nutrition, as well in pharmaceutical applications. From the metabolic point-of-view, the pathway taken by MCTs and LCTs differed in terms of the route taken after digestion and their metabolism. Generally, MCT is favoured over LCT due to its potential to accelerate transit time in the gastrointestinal tract (GIT) and imposing a lower physiological burden to be metabolised [51]. Therefore, MCT is preferred in parenteral drug delivery and drug formulation, imposing a lower metabolic demand in delivery drugs. The higher rate of MCT hydrolysis, compared to LCT, proved that the metabolism of MCTs is faster than LCTs. This is proven in another study where MCT and LCT were compared based on the level detected in non-cannulated and cannulated rat lymph. The detected LCT concentration was higher in the lymphatic system compared to MCT, acknowledging that LCTs require prior packaging and are subject to transportation through the lymphatic system from the GIT and synthesised into triglyceride for storage [52,53,54]. Meanwhile, MCTs are rapidly catabolised into FAs in intestinal epithelial cells and transported directly into the portal vein, bypassing the lymphatic system and blood circulation before undergoing oxidation in the liver and, finally, stored in the adipose tissue [52]. The transportation of MCTs and LCTs exhibit a different degree of absorption rate from the intestine before they reach saturation. A study reported that the time required to reach maximal absorption of chain-triglycerides is proportionate to the length of the chain [55]. Therefore, it is understood that the absorption rate of MCT is shorter compared to LCT. Despite this, LCT showed a higher percentage in terms of systemic drug distribution. This is due to the reason that LCT required the formation of a complex with micelle before being transported through the lymphatic system and blood circulation. This allows a slower rate of absorption and higher volume of drug distribution along the systemic circulation. This is supported in a study to elucidate the behaviour of LCT and LCT/MCT mixture in a formulation of bupivacaine emulsion [56]. In this study, LCT increased the distribution of bupivacaine throughout the system. However, the drug-loaded LCT exhibited higher elimination rates compared to LCT/MCT mixture and was shown to reduce the drug concentration in plasma more effectively. This is explained by the higher elimination rate by the liver due to the higher volume of blood circulation carrying LCTs through hepatic artery compared to MCTs, which are transported via the portal vein. Regarding palm oil and its fraction, it can be deduced that FAs from CPKO, RBD PKO, RBD PKL and RBD PKS would be digested at a higher rate compared to CPO and other fractions as the former contain primarily MCTs. 

Dietary supplement, parenteral nutrition and lipid-drug formulation have incorporated MCTs and LCTs in their applications. Among the benefits or characteristics of MCTs, apart from LBFs and other applications stated above, are anti-inflammatory properties [57], reducing oxidative stress in cardiac remodelling [58] and promoting allergy sensitisation and anaphylaxis in animal models [59]. MCTs have been proven as an effective fat emulsion in parenteral nutrition as they imposed less burden to physiological and metabolic demand in human GIT in providing daily dietary caloric requirement, especially in individuals with digestive tract diseases such as cancer [60] and fatty acid metabolic disorder [61]. In the pharmaceutical industry, MCT and LCT are heavily considered as part of drug formulation from which both are selected as drug carrier and as an excipient in LBF to promote better digestibility and drug distribution. 

## 4. Lipid-Based Formulation

Lipid-based formulations (LBFs) have attracted considerable attention as well as played an emergent role in the pharmaceutical industry as effective ways to improve oral bioavailability of lipophilic compounds. The LBF is designed for improved oral absorption in such a way that it tends to generate and maintain a supersaturated drug concentration in vivo. This approach is achieved through dissolving the hydrophobic drug in LBF, emulsifying the oil phase in water, and maintaining the drug in the solubilised state in the GIT until absorption has occurred. Thus, the lipid excipients for the formation of emulsions play the most critical role in the formulation performance. The formulation of LBF can be in the form of a simple oil solution to complex mixtures of oils, surfactants, co-surfactants and co-solvents [62]. Over time, new LBFs are being created and tested with different ratios between the ingredient and compounds. Through extensive studies and an understanding of LBFs and their drug delivery system (DDS), it is possible to address the ideal method and form of delivery [63] and metabolic pathway taken in order to express better biocompatibility and versatility [64]. 

LBFs are classified based on their specific parameters and categorised into four types, called Type I, II, IIIa and IIIb [65]. The hydrophilic content in the formulation increases from Type I to Type IIIb LBF, whereby Type I formulations are 100% oil solutions, which exhibit poor aqueous dispersions but are able to be rapidly digested and absorbed in the GIT. Type II formulations are often referred to as self-emulsifying drug delivery system (SEDDS), consisting of oils and water-insoluble surfactants; subsequently, they form fine emulsions when introduced into aqueous media. Self-emulsifying formulations have the advantage of being able to self-emulsify and spread readily in the GIT. Type III formulations are SEDDS that disperse well and have been further modified from self-microemulsifying (SMEDDS) to self-nanoemulsifying (SNEDDS) drug delivery system. SNEDDSs are classified as Type IIIa or IIIb, with both containing one or more co-surfactant or hydrophilic co-solvent. However, the difference in these two types is based on the ratio of hydrophilic co-solvent incorporated into the formulation. Type IIIb contains a higher ratio of co-solvent compared to Type IIIa. Digestion and emulsification in both SMEDDS and SNEDDS are not required as these types of formulations are able to self-emulsify effectively, producing particles with a small-enough size to be readily absorbed in the GIT.

An LBF must be studied and optimised before being tested to identify the behaviour of the formulation and interaction between excipients and other active compounds. Among popular excipients favoured are those from peanut, olive and soybean, in the form of oil to emulsify a drug. Palm oil exhibits the same capability to emulsify drug and as a drug carrier for systemic delivery. Studies conducted on PKOE showed that the lauric acid contained in it appeared to have similar characteristics to Tween 20 surfactant, a type of synthetic oil safe for consumption that is commonly used as an excipient in LBF. From the study, PKOE appeared to mix ideally, producing stable formulations and forming micelles similar to the formulations using Tween20 [28].

The pharmaceutical industry and drug manufacturers favour LBF because of improved drug-loaded bioavailability profile [66,67,68], safeness for consumption [69], a good profile of drug absorption [70], protection provided to the loaded drug in formulation [71], reducing food effect [72] and promoting high drug payload despite its low dose formulation [73]. On the contrary, the disadvantages of LBF include oxidation of excipient, specific storage requirement, higher frequency of drug intake required compared to solid dosage form and, to some extent, GIT-adverse effects [74,75]. Among the latest Federal Drug Administration (FDA) approved LBF drugs are calcifediol [76], nintedanib [77], enzalutamide [78], isotretinoin [79] and loratadine [80]. 

## 5. Drug Delivery System of a Lipid-Based Formulation Using Palm Oil

**Figure 3:**
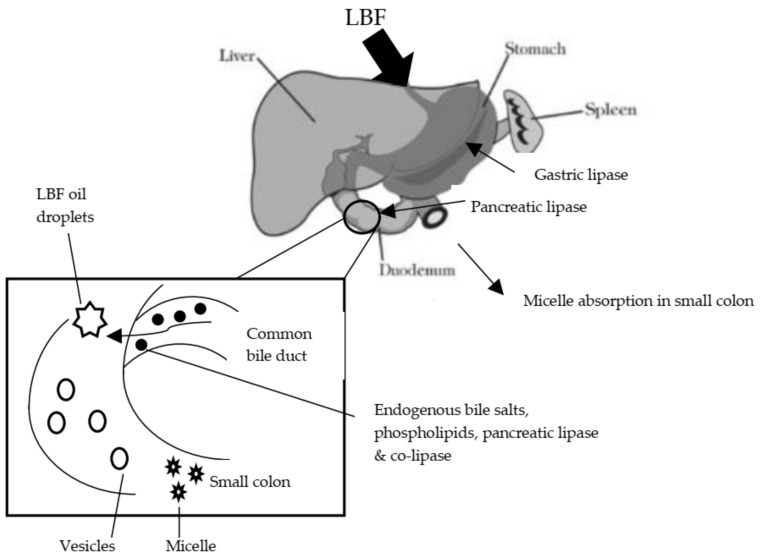
Diagram of drug delivery system for oral lipid-based formulation (LBF) drug and its absorption. LBF drug enters the stomach where gastric lipase hydrolysed and stabilised the drug in the stomach, due to the presence of its excipient and surfactant. An excipient is defined as the drug carrier, usually lipids that help to solubilise drugs. A surfactant is a surface-active agent that solubilises drugs further or emulsifies them. Gastric agitation and emptying transport these hydrolysed lipids into the duodenum, where they are subjected to bile salt and pancreatic lipase digestion. Subsequently, micelles are formed and loaded with an LBF drug. Formed micelles are transported along the small intestine where absorption occurs, and the drug is transported to the targeted organ for drug loading.

An optimised LBF is incomplete without applying the drug delivery system to determine the form, strategies and method in delivering a drug formulation. The drug delivery system ensures a drug formulation is delivered effectively and safely. This system consists of many classes and classification based on the form and mechanism of drug delivery. For instance, a drug is designed in the form of topical application, such as cream and gel, while some other drugs are designed for oral route and parenteral formulation. The oral dosage form is the most common form of a drug formulation. This covers LBF [81]. LBF drug delivery systems apply various forms and mechanisms to ensure the incorporated lipophilic or hydrophobic drug is absorbed in the GIT and reaches the target organs. As described above, LBF includes triglycerides, surfactants and co-solvents, which can be grouped as excipients that assist drug absorption. The objective of oral LBF is to ensure drug solubility in the GIT and prevent precipitation. The introduction of LBF consisting mainly of lipids triggers the GIT physiological response by the secretion of lingual (from oral cavity) and gastric lipase hydrolysing TAG in the stomach. From here, the stomach promotes lipid emulsification by gastric agitation and an emptying process. Excipients such as palm oil, especially MCTs, appear lipophilic and contain drug solvent capacity; hence, they are able to promote stabilisation of the hydrolysed TAG. Co-solvent, usually added together in Type II and Type III LBFs, promotes the dissolution of the drug by avoiding precipitation in the stomach [62]. The emulsified and hydrolysed LBF is then transported into the duodenum, stimulating the release of bile salts and pancreatic juice (lipase and co-lipase), thus enabling the formation of micelles and occurrence of absorption in the small intestine [82,83]. Figure 3 below illustrates the drug delivery system of oral LBF and how it is absorbed and transported to organs.

### 5.1. Emulsion, Microemulsion and Nanoemulsion

Type I formulation contains 100% TAG or mixed glycerides without the use of surfactant and co-solvent. The particle size of dispersion appears coarse and is of limited importance in aqueous solutions. However, Type I relies heavily on the digestibility by bile acids and enzymes when it is introduced in the GIT to be emulsified and dispersed for absorption. The drug delivery system of Type I classification includes emulsion, microemulsion and nanoemulsion. Refined palm oil and POE are both commonly selected as a surfactant in Type 1 formulation. An emulsion is defined as a colloidal system requiring dispersion, and the formulation contains more than one immiscible liquid and is mixed and stabilised through the addition of one or more surfactants [84]. The microemulsion commonly appears as a clear and stable emulsion with an isotropic mixture of oil, water and surfactant, frequently in combination with a co-surfactant [85]. The term microemulsion is defined by the droplet size formed, ranging between 20 and 200 nm after being emulsified in the GIT [86]. On a smaller scale, the nanoemulsion is defined as a thermodynamically stable isotropic and clear dispersion of two or more immiscible liquids, and is stabilised by co-surfactant producing droplet sizes ranging from 100 to 600 nm [63]. The definition of nanoemulsion varies between authors regarding the droplet size. However, a mutual agreement is made based on the properties of nanoemulsion, whereby it should appear as being thermodynamically stable and possess long-term physical stability [86,87]. Droplet size formed by emulsion, microemulsion and nanoemulsion is influenced by the concentration of oil and drug in a formulation [87], suggesting that optimisation of a formulation is imminent in consideration of the objective and purpose of the formulation.

The use of palm oil, including its fractions such as TAG, remained limited. Other natural oil sources such as castor oil and soybean oil have been selected and used extensively as the oil phase in LBFs in previous studies [67,68,72,88]. However, there were studies in which palm oil was used as an excipient in Type I LBFs and the drug formulations showed improved bioavailability and permeability. As an example, a study had formulated and tested a topical drug delivery of betamethasone 17-valerate emulsion using RBD PL as the oil phase [84]. This study was carried out to compare the formulation to the commercially available emulsion of betamethasone containing methanol. The result from the study showed higher bioavailability of the topical emulsion containing RBD PL (4.76 times higher) based on in vivo drug release using cellulose acetate membrane. Better bioavailability was contributed to by RDB PL through enhancement of the formulation’s stability in resisting the movement of oil globules. Apart from that, the formulation exhibited higher permeability as betamethasone-17 showed higher affinity for RBD PL compared to methanol or other mixtures. The formulation containing RBD PL as a vehicle was detected to degrade less than 4% during the period of three months as the formulation was subjected to different temperatures. Therefore, selecting RBD PL in LBF emulsions as the vehicle for topical drug delivery showed promising capabilities through producing superior permeability, better bioavailability and longer drug shelf-life. 

Lipid-based formulations involving palm oil have been shown to exhibit stable formulations for drug delivery. In a study of microemulsion formulation using palm oil, Lee et al. [89] formulated lipid oil-in-water emulsions using PKO, coconut kernel oil (CKO) and soybean oil (SBO) blended and loaded with phenytoin. The study detected that mixing the formulation with surfactant Tween 80 at an oil-to-surfactant ratio of 1:9 produced 100% emulsion stability. However, blended CKO and SBO microemulsion hydrogels were regarded as the highest potential candidates for topical phenytoin delivery due to achieving highest drug release rate (93.21%) in 12 h, compared to PKO. Despite this outcome, the utilisation of PKO should not be neglected, as the potential of PKO as a good excipient candidate was comparable to both CKO and SBO as shown in the study. Palm kernel oil itself has exhibited its ability to stabilise an emulsion formulation, as well as producing droplet sizes lower than 50 nm—comparable to CKO without mixing with a surfactant. Therefore, the mixture of PKO with other excipients and surfactants may be able to boost the stability of an emulsion and promote effective drug delivery. 

Lipid-based formulations based on palm oil could be manipulated to target specific organs and have demonstrated versatility concerning the dosage form. For example, the findings of Zainol et al. [10] suggested an improved formulation of palm-based nanoemulsion system containing levodopa. Their study was carried out to establish the potential of a nanoemulsion system containing levodopa to travel across the blood–brain barrier, allowing the treatment of Parkinson’s disease. The formulation consists of a palm oil mixture containing MCT, lecithin and Cremophor EL. This formulation exhibited stability and good shelf-life after storing at 4 °C for up to six months, suggesting similar findings as the study mentioned above. The outcome from their study also showed that due to the size, the nanoscaled formulation was able to cross the blood–brain barrier. In another similar study, a PKOE nanoemulsion was formulated and loaded with chloramphenicol to treat meningitis [90]. Nanosized particles were achieved in the study, exhibiting the potential to cross the blood–brain barrier as well during in vivo drug delivery. A study by Alayoubi et al. [91] investigated a similar means of drug delivery system but the author’s drug of interest was the tocotrienol-rich fraction (TRF) of palm oil as an anticancer formulation targeting the brain. Their study showed similar findings in terms of palm oil LBFs capable of crossing the blood–brain barrier. Both studies by Musa et al. [90] and Alayoubi et al. [91] formulated nanoemulsion-loaded drugs in the form of a parenteral drug delivery system, suggesting that the versatility of palm oil LBF dosage is not limited to the oral route but is also applicable to other forms of delivery system. The drug delivery system (DDS) of nanoemulsion is not only limited to the brain, but a study was done to deliver docetaxel-loaded nanoemulsion in which PKOE was included as part of the formulation to be delivered to the lungs [92]. The nanoemulsion exhibited high thermal and colloidal stability, as well as was highly selective towards human lung carcinoma cells rather than normal cells, suggesting a highly specific drug-targeting system posed by an LBF, especially involving a palm oil-based formulation. Similarly, an LBF using palm oil was constructed for a topical application using POE loaded with ibuprofen [93]. An ultrasound-activated emulsification using PL has been studied as well [94]. Both studies described effective drug delivery. However, results from the studies described above were in the in vivo stage. Clinical trials on these drugs should be carried out to exhibit their full potential. Studies involving palm oil in Type I LBFs are summarised in Table 2 below, to provide an overview of the formulations and the drugs incorporated, as well as the dosage form. 

Given the above, Type I LBFs consisting of palm oil presented benefits primarily in stabilising formulations and enhancing drug absorption and bioavailability. Palm oil fractions, such as PKOE and PKO, containing MCTs allow the formulation to be in all types of emulsion. The drug delivery system of palm oil LBF is specific and effective, especially in crossing the blood–brain barrier or regarding their drug loading capacity for their use in cancer treatment. Apart from that, the studies above showed that using palm oil as an excipient is comparable to other commonly used natural oils, such as soybean and peanut oil, with a proven versatility and flexibility of palm oil in LBFs, thus showing a promising outcome. 

### 5.2. Self-Emulsifying Drug Delivery System

Type II formulations are categorised as a self-emulsifying drug delivery system (SEDDS). SEDDS generally contain about 40% to 80% TAG or mixed glycerides with a mixture of 20% to 60% surfactants. This is to improve the solvent capacity, which further allows better emulsification and digestibility. Co-solvent is not included in this type of formulation. The particle size produced is, at an average, between 100 to 250 nm. Aqueous dilution would not affect the solvent capacity of SEDDS and although the requirement of digestibility is not vital, it is likely to occur. SEDDS is a more complex system when compared to the emulsion. Self-emulsification was first defined by Wakerly et al. [105] as emulsification formed from oil and water through weak mechanical shear forces. SEDDS formulation includes a lipophilic phase and drug, with or without surfactant. At present, SEDDS formulation consists of a mixture of lipid excipient and non-ionic surfactants without the need of water forming transparent isotropic solutions [106]. As an example, Miglyol 812, first used by Wakerly et al. [105], consisted of saturated coconut esters and MCT (caprylic and capric fatty acids) derived from PKO. The study showed that self-emulsification occurred within the GIT with the use of surfactant oil solutions such as Miglyol 812, thus allowing the use of soft gelation-encapsulated formulation for effective drug delivery. This study and many other similar studies [107,108] paved the road for self-emulsification drug delivery systems. The formulation of SEDDS, especially excipients, are not taken as a whole but rather in the form of FAs. Therefore, the length of FAs, such as LCTs and MCTs, play a major role in deciding the behaviour and interaction with surfactant in a formulation.

LCT- and MCT-containing oils have been utilised in designing SEDDS formulation. Palm oil and PKO, containing LCT and MCT, respectively, have been shown to provide effective drug delivery in SEDDS as excipients. A SEDDS formulation involving metronidazole was tested using both PO and PKO. The result from the study showed quick drug release and both oils offered stable formulation when blended with metronidazole, resulting in the absence of precipitation [107]. Apart from that, palm oil FA in SEDDS formulation provides enhanced capacity and permeability. As an example, a formulation of fluoroquinolones antibiotic ciprofloxacin was developed in targeting lungs affected by cystic fibrosis (CF) [109]. This study utilised a selection of oleic acid, Capmul MCM EP, Miglyol 840 and Peceol in the oil phase. These oils were combined with Labrafil M1944 CS, Capmul PG8, Cremophor EL, Labrasol or Tween 20 as surfactants, and PEG 400, PG or Transcutol as co-solvent. The outcome of the study showed oleic acid to be the ideal solvent for SEDDS formulation in delivering ciprofloxacin effectively. The droplet size of 25 nm was developed in combination with Labrafil, Cremophor and Labrasol as surfactants and Transcutol as co-solvent. This formulation exhibited enhanced capacity in permeating porcine intestinal mucus and CF sputum in the in vivo studies, with improved antimicrobial activity due to the effective membrane effect of drug release. Oleic acid, caprylic and capric fatty acids are MCTs from PKO exhibit the same potential as palm oil fractions in their use in developing effective SEDDS formulations with efficient drug release and increased bioavailability, as demonstrated in the studies above. 

Using palm oil in SEDDS is not limited to its FA but also includes vitamin Es, such as tocotrienol and tocopherol, that were used as active ingredients in the formulation. In another study, the tocotrienol-rich fraction (TRF) of palm oil was utilised as the oil phase of the formulation containing 30% α-tocopherol and 70% α, γ and δ-tocotrienols [110]. TRF was mixed with Tween 80 or Cremophor EL as the emulsifier to determine the optimal formulation. The addition of active ingredient in the form of palm oil vitamin E provides benefits to the formulation. In this case, γ-tocotrienol was bound tightly to the hydroxyl group of Cremophor EL, while in Tween 80 it was bound to the head group of the water/emulsion interface. Binding of γ-tocotrienol to the hydroxyl group allows partitioning of TRF within Cremophor micelles, resulting in a stable structure instead of binding to polar heads, such as seen with Tween 80. Studies have remained limited when it comes to the utilisation of palm oil content in SEDDS development. However, the studies above have demonstrated that using palm oil in SEDDS is able to provide rapid drug release formulation, high permeability and promote a positive effect on surfactants and other components in SEDDS. 

In summary, SEDDS relies heavily on the utilisation of MCT and LCT as part of its formulation. The advantage in SEDDS using palm oil is that the fruit and kernel consist of both LCTs and MCTs respectively, resulting in the efficiency of choosing readily available FAs to be incorporated in the formulation. On top of that, palm oil MCT promotes SEDDS-enhanced permeability and effective drug delivery, as well as stabilising the formulation especially when SEDDS involves surfactants. However, readily available commercial MCT and FAs from palm oil should be produced. This is to provide a wider variety of options in designing SEDDS with palm oil fractions as one of the ingredients, along with the benefits described above. 

### 5.3. Self-Microemulsifying Drug Delivery System

The self-microemulsifying drug delivery system (SMEDDS) is the Type III LBF. Compared to SEDDS, SMEDDS has smaller lipid droplet size, ranging from 200 nm to 5 mm [65]. Apart from the size, SMEDDS formulation requires the use of both surfactant and co-surfactant to generate microemulsions, and the emulsions generally appear as clear fluid. The utilisation of a palm oil component has been applied in developing SMEDDS formulation and provides effective drug delivery in many dosage forms. As an example, an antiviral agent against HIV-1 using SMEDDS was developed for intravaginal delivery [111]. The oil phase of the formulation includes Capmul MCM (mono/diglyceride of caprylic acid from PKO), Capmul PG-8, Captex 200 (mixed diesters of caprylic/capric acids on propylene glycol) and Miglyol 812N (an MCT). Surfactants utilised in this study were PEG 300, Tween 80 and Cremophor RH 40. SMEDDS should appear clear and as a monophasic liquid at ambient temperature to prevent drug precipitation, which this study was able to achieve. The ratio between oil, surfactant and co-surfactant play significant roles in determining the efficiency of drug release. From this study, 10% (*w/w)* Capmul MCM for the oil phase, 81% (*w/w*) Cremophor RH 40 as a surfactant and 9% (*w/w*) PEG 300 as the co-surfactant exhibited improved release of the drug compared to the powder form of the drug. 

A study was carried out to understand the particle size and phase behaviour when palm MCT was used in developing SMEDDS combined with non-toxic surfactant [112]. Miglyol 812 and Imwitor 988 were selected as MCT oils and mixed with a surfactant, Tagat TO. The self-emulsification resulted in the dispersion of submicron particles less than 50 nm. From this study, the concentration of surfactant from 20% to 45% showed improved stability of submicron emulsions. Palm oil in the SMEDDS formulations shown in the studies above offers stability when combined with surfactants and allows effective drug release in various forms of delivery systems. 

## 6. Future and Current Perspectives of Palm Oil in Lipid-Based Formulations

The role of palm oil in LBF and DDS are seen to be extensive, yet not highly appreciated and recognised. Type I, II and III LBFs, as described above, have been in existence for some time [21]. Despite that, the utilisation of palm oil in LBFs appeared to be restricted to Type I compared to Type II and III LBFs. The reason for this outcome is due to the fairly simple preparation of using palm oil as a whole, without the addition of surfactant and co-surfactant in Type I LBFs compared to Type II and III. The formulation of Type II and III LBFs is more complex and difficult, requiring extensive tests in predicting its behaviour as these classes have high hydrophobic content. The other reason is likely due to the lack of studies advocating the exploitation of palm oil in LBF formulations compared to other readily available natural oils, such as olive, corn and castor oil. Putting the focus on Type II and Type III classes and related studies, the excipients required were mainly a combination of several oils and specific active compounds from natural oil, rather than using them as a whole. The compounds from palm oil such as MCTs, olein, caprylic and capric fatty acids were selectively chosen to be combined with other surfactants in Type II and III formulations. Again, investigation and exploitation of palm oil compounds are lacking, resulting in fewer LBFs using palm oil compared to other vegetable and natural oils. New formulations are still being created and tested with many of the current drugs being mostly lipophilic and hydrophobic. Greater focus and further investigation should be carried out in choosing palm oil contents as the candidates in LBFs, especially drug-loaded micro- and nanoemulsions, SEDDS and SMEDDS. At present, self-nanoemulsifying drug delivery system (SNEDDS) has been under heavy investigation for the development of new drug formulations with a highly effective and efficient drug delivery system. Other forms of LBFs have emerged, providing promising results such as solid lipid particles (SLPs) [113], nanostructured lipid carriers (NLCs) [114], solid dispersions and nanocapsules. Nonetheless, despite limited studies, palm oil has been utilised in solid dispersion [115] and nanocapsule formulations [116]. Both studies have elicited positive results in terms of shelf-life, stable formulation, high drug-loading capacity and bioavailability. Yet, these studies remained limited.

## 7. Conclusions

This review highlighted the imperative positive outcomes of using palm oil in LBFs. First, palm oil contains extensive phytonutrients such as vitamin E and carotenes in its fruit and seed. The oil produced from fruit and kernel has a good safety profile suitable for consumption. Palm oil and PKO contain LCTs and MCTs respectively, making the yield easily exploitable and readily available for any forms of formulation and function, depending on the objective of the drug design in LBF. Apart from improved bioavailability and safety profile of using palm oil in LBF, these formulations are proven to exhibit high drug-loading capacity and providing efficient drug absorption due to their property of high permeability. In addition, palm oil LBFs have been shown to be suitable candidates for any type of formulation, dosage form and purpose. The FAs and oils are unaffected by other surfactants and excipients when mixed in a formulation, but rather the drug performance and stability were enhanced during delivery. All formulations using palm oil and its fractions offered long shelf-life, making palm oil LBF a great choice in formulating new drugs, which are often hydrophobic and lipophilic.

## Figures and Tables

**Table 1:** The major compositions and percentages of fatty acids in palm oil and its fractions.

Palm Oil Fractions	Major Composition	Percentage (%)	Reference
Crude palm oil	Palmitic acid	46	[29]
	Stearic acid	3	
	Oleic acid	40	
	Linoleic acid	9	
Refined palm oil	Palmitic acid	44	[49]
	Oleic acid	39	
	Linoleic acid	10	
Palm olein	Oleic acid	43	[38]
	Palmitoleic acid	41	
	Linoleic acid	11	
Palm stearin	Palmitoleic acid	57	
	Oleic acid	40	
Super olein	Oleic acid	47	[39]
	Palmitic acid	35	
	Linoleic acid	14	
Palm mid-fraction	Palmitic acid	58	[40]
	Oleic acid	32	
Crude palm kernel oil	Lauric acid	50	[33]
	Myristic acid	17	
	Palmitic acid	9	
	Oleic acid	17	
Refined palm kernel oil	Lauric acid	48	[44]
	Myristic acid	16	
	Oleic acid	15	
Palm kernel olein	Lauric acid	46	[45]
	Oleic acid	18	
	Myristic acid	14	
Palm kernel stearin	Lauric acid	56	[47]
	Myristic acid	21	
Palm kernel oil ester	Oleyl laurate	54	[42]
	Oleyl myristate	14	
	Oleyl oleate	6	
	Oleyl palmitate	6	

**Table 2:** Summary of Type I LBF using palm oil. Refined–bleached–deodorised palm olein (RBD PL), palm kernel oil (PKO), refined–bleached–deodorised palm oil (RBD PO), red palm oil (RED PO), palm kernel oil ester (PKOE), palm oil ester (POE) and palm olein (PL).

Compound/Drug	Drug Category	Dosage Form	Oils	Surfactant	Co-Surfactant	Reference
Betamethasone 17-Valerate	Topical emulsion	Oral	RBD PL	Span20/Tween 20 mixture	-	[84]
Phenytoin	Microemulsion	Oral	PKO	Tween 80	-	[89]
Levodopa	Nanoemulsion	Oral	RBD PO & MCT	Cremophor EL	-	[10]
Genistein	Nanoemulsion	Topical	Red PO	Solutol and vitamin E blended	-	[95]
Paclitaxel	Nanoemulsion	Oral	RBD PO	Vitamin E TPGS	-	[6]
Aripiprazole	Nanoemulsion	Oral	PKOE	Tween 80	-	[96]
Chloramphenicol	Nanoemulsion	Oral	PKOE	Safflower seed oil	Tween 80	[90]
Diclofenac acid	Nanoemulsion	Topical	POE	Tween 80	-	[97]
Sodium diclofenac	Nanoemulsion	Oral	PKOE	Cr EL	-	[98]
Ibuprofen	Nanoemulsion	Topical	POE	Carbopol 940	-	[93]
Ibuprofen	Nanoemulsion	Topical	PKOE	Tween 80	-	[99]
Vitamin E	Nanoemulsion	Topical	PL	Brij 30	-	[100]
β-d-glucan	Nanoemulsion	Oral	PL	Kolliphor RH40	-	[94]
β-Carotene and Tocopherol	Nanoemulsion	Oral	PL	Lecithin	-	[101]
Ketoprofen	Nanoemulsion	Topical	POE	Tween 80	-	[102]
Docetaxel	Nanoemulsion	Nebulizer	PKOE	Tween 80	Span 80	[92]
Tocopherol Acetate	Nanoemulsion	Topical	POE	Tween 80	Pluronic F-68	[103]
Tocotrienol Rich Red Palm Oil	Nanoemulsion	Oral	Red PO	Tween 80	Span 80	[104]
Tocotrienol	Nanoemulsion	Topical	POE	-	-	[41]
